# Empowering Men to Take Control of Their Own Health: Development and Validation of the Men’s Response to Colorectal Cancer Screening Scale (MR–CCSS)

**DOI:** 10.3390/healthcare13121433

**Published:** 2025-06-15

**Authors:** Vesna Jašić, Mirko Prosen, Sabina Ličen

**Affiliations:** 1 Institute of Oncology Ljubljana, Zaloška cesta 2, 1000 Ljubljana, Slovenia; vjasic@onko-i.si; 2 Department of Nursing, Faculty of Health Sciences, University of Primorska, Polje 42, 6310 Izola, Slovenia; mirko.prosen@fvz.upr.si

**Keywords:** colorectal cancer screening, men’s health, health behaviour, health education, gender equity, preventive health, health promotion

## Abstract

Background/Objectives: Despite the proven effectiveness of colorectal cancer screening, men are less likely to participate than women, with emotional, behavioural and informational barriers contributing to this disparity. The aim of this study was to develop and validate the Men’s Response to Colorectal Cancer Screening Scale (MR–CCSS), a gender-sensitive instrument for measuring key factors that influence the participation of men in colorectal cancer screening. Methods: The scale was developed through a structured process that included qualitative data from focus groups (*n* = 20 men) and expert review (*n* = 11 professionals). Initial item pools were refined based on indices of content validity (I-CVI ≥ 0.78; S-CVI/Ave ≥ 0.90), resulting in a 23-item scale. The MR–CCSS was administered to 289 Slovenian men aged 50–74 years, and its psychometric properties were assessed using exploratory factor analysis, confirmatory factor analysis and reliability tests. Results: The EFA revealed a five-factor structure. Together, these factors explained 61.9% of the total variance. The CFA confirmed the robustness of the model (CFI = 0.928, TLI = 0.910 and RMSEA = 0.056). The internal consistency was acceptable to good, with Cronbach’s alpha between 0.665 (factor 4) and 0.833 (factor 5) for the subscales and 0.863 for the total scale. The ROC analysis showed moderate predictive accuracy (AUC = 0.702), with an optimal cut-off value of 92.5 (sensitivity = 0.782 and specificity = 0.509) for participation in screening. Conclusions: The MR–CCSS is a valid and reliable tool for identifying barriers to colorectal cancer screening in men. Its use can serve as a basis for gender-specific interventions, customised health education and strategies to improve screening equity.

## 1. Introduction

Colorectal cancer is one of the most common and deadliest cancers worldwide and represents a major public health challenge due to its high incidence, mortality and long-term burden on healthcare systems. In 2020 alone, more than 1.9 million new cases and around 930,000 deaths were reported worldwide [1]. The potential for early detection and treatment has led many countries to introduce population-wide screening programmes targeting people in specific age groups, usually between the ages of 50 and 74. These programmes, which often use faecal occult blood tests (FOBT) or colonoscopy, are in line with European Union recommendations on cancer screening [2].

Slovenia has adopted such an approach with its national colorectal cancer screening programme Svit, in which all eligible residents are systematically invited to participate every two years using home stool sample kits. Although the programme has proven effective in reducing incidence and mortality, participation rates remain suboptimal, particularly among men [3]. This is worrying as there is clear evidence that participation in screening improves both quality of life and survival [4,5]. Before the Svit programme was introduced, colorectal cancer in Slovenia was often diagnosed at an advanced stage, which led to poorer treatment outcomes. Since then, the programme has made remarkable progress, with 70% of diagnosed cases now being detected at an early stage [3].

### 1.1. Background

While men are often perceived as holding positions of strength and privilege, global health data reveals that they face significant health disadvantages. Across the world, men have shorter life expectancies, higher rates of premature mortality and poorer health outcomes than women [6]. These disparities are not only the result of biological differences but are strongly influenced by social determinants of health and culturally embedded gender norms. For example, masculine ideals such as stoicism, independence and emotional reserve often discourage men from seeking help and reduce the likelihood that they will utilise preventive health services [7]. In times of social or economic change, men’s vulnerability tends to increase as their health is not only characterised by occupational stress and lifestyle, but also by their access to and utilisation of health services, which is often suboptimal [8,9]. Healthcare systems and public health strategies have historically under-recognised the health needs of men, resulting in a lack of tailored health education and support services for this population.

To understand these patterns, researchers have increasingly turned to theories of health behaviour. Socially constructed gender roles, i.e., the ways in which societies define masculinity and femininity, play an important role in shaping individual attitudes and responses to health interventions [10]. These roles are relational and dynamic and are influenced by social class, education, ethnicity and age. They can influence risk perception, willingness to act and willingness to participate in organised health programmes such as cancer screening [11]. In addition to these psychosocial influences, structural barriers such as health literacy, cultural beliefs, logistical challenges and communication deficits can also prevent men from accessing or responding to screening [12]. Health literacy, defined as the ability to obtain, process and understand health information, has been shown to be a critical determinant of health behaviour and a key factor in promoting active participation in one’s healthcare [13]. Low health literacy among men is associated with reduced screening uptake, poorer disease management and less informed health decisions.

Despite the clear need, health education programmes still lack gender sensitivity. Many are developed generically without addressing the specific needs, motivations and communication preferences of men [14]. Public health messages that are not aligned with men’s identities and life contexts can reinforce disinterest rather than promote positive action [15].

### 1.2. Aim of the Study

The aim of this study was to develop and validate the Men’s Response to Colorectal Cancer Screening Scale (MR–CCSS), a gender-sensitive instrument designed to assess men’s attitudes, beliefs and perceived barriers to colorectal cancer screening, with the objective of identifying the key factors that influence their participation.

## 2. Materials and Methods

### 2.1. Scale Development 

The development of the Men’s Response to Colorectal Cancer Screening Scale (MR–CCSS) was carried out in 2023 in a structured and systematic process. This process included several key phases: (a) the creation of an initial set of items and a corresponding response scale, (b) the assessment of the content validity of the scale and (c) the performance of tests to assess the factor structure and reliability of the final scale.

#### Item Generation Based on Qualitative Data 

The initial pool of items (*n* = 64) was generated based on the results of a qualitative study conducted in 2022. This study used a semi-structured interview approach with three focus groups consisting of 20 male participants aged between 50 and 74 years, all of whom were eligible for colorectal cancer screening in Slovenia. The aim was to investigate the cognitive, emotional, social and cultural factors that influence men’s attitudes towards colorectal cancer screening.

The data was analysed using a thematic analysis, resulting in the identification of four key themes. These themes reflected men’s perspectives and experiences related to colorectal cancer screening participation. The main themes included (1) experiences with healthcare, particularly men’s general approach to health and help seeking; (2) knowledge, attitudes and beliefs about the Svit screening programme; (3) perceptions and emotional responses to the screening process itself; and (4) the influence of the social environment on screening responsiveness [16]. These findings provided valuable context for generating scale items that reflect the cognitive, emotional and social dimensions of men’s responsiveness to colorectal cancer screening.

The thematic framework served as a conceptual basis for the development of the items. For example, codes relating to embarrassment during screening were translated into statements measuring emotional discomfort, while themes relating to masculinity and denial of vulnerability were incorporated into items measuring health-related identity. This inductive, data-driven approach ensured that the scale was based on the lived experiences and culturally embedded beliefs of the target population.

To ensure content coverage and conceptual clarity, each topic was mapped to one or more suggested items. All items were formulated as declarative statements in simple and clear language suitable for the age group of the target population. A five-point Likert scale was chosen for the responses (1 = strongly disagree to 5 = strongly agree), which is based on proven methods for measuring health attitudes [17].

### 2.2. Pilot Study

A pilot study was conducted to evaluate the clarity, relevance and comprehensibility of the preliminary 64-item version of the MR–CCSS. This step served as an essential foundation for subsequent phases of content validation and psychometric testing of the scale.

#### 2.2.1. Content Validity of the Newly Developed Scale

A panel of 11 experts was recruited to assess the content validity of the preliminary version of the MR–CCSS. The panel consisted of nursing, public health and health promotion professionals, including 7 women and 4 men aged between 32 and 53 years (M = 42.3, SD = 6.6). All experts had more than five years of professional experience in their respective fields.

Each item from the initial pool was independently rated for relevance using a four-point ordinal scale (1 = not relevant to 4 = very relevant) and for clarity using a three-point scale (1 = not clear to 3 = very clear). Based on the experts’ ratings, indices of content validity at item level (I-CVI) were calculated for each item and an index of content validity at scale level (S-CVI/Ave) was calculated as the average of all I-CVIs.

In accordance with established guidelines [18,19], an item was considered acceptable if it achieved an I-CVI of ≥ 0.78. The overall scale was considered to have satisfactory content validity if the S-CVI/Ave was ≥ 0.90. Items that did not meet these thresholds were excluded from further analysis or subjected to revision. To complement the CVI analysis, Kendall’s W was used to assess the degree of agreement among experts.

#### 2.2.2. Construct Validity and Reliability

To assess the construct validity and internal consistency of the MR–CCSS, a multi-stage psychometric evaluation was conducted. This comprised an exploratory factor analysis (EFA), followed by confirmatory factor analysis (CFA) using structural equation modelling (SEM) and a reliability assessment using Cronbach’s alpha.

The EFA was used to identify the factor structure and to reduce the items without losing conceptual relevance. The suitability of the data was confirmed using Bartlett’s Test of Sphericity (*p* < 0.05) and the Kaiser–Meyer–Olkin (KMO) measure, with values ≥ 0.60 considered adequate and values > 0.80, indicating excellent sampling adequacy. An EFA was performed using principal axis factoring with Promax rotation, and factors were selected based on eigenvalues > 1.0, the scree plot and parallel analysis. Items with loadings < 0.40 or high cross-loadings were removed.

The factor structure determined in the EFA was then tested with the CFA. The model fit was assessed using standard indices: The *χ*2/df ratio (< 3 acceptable), CFI and TLI (≥ 0.90 for good fit), RMSEA (≤ 0.08) and SRMR (≤ 0.08) [20]. Where necessary, modifications were guided by modification indices. SEM allowed for additional insight into the relationships between latent variables and observed indicators, supporting construct validity.

Internal consistency was examined using Cronbach’s alpha. Values ≥ 0.70 were considered acceptable, and those > 0.80 indicate good reliability [21].

### 2.3. Sample

The pilot study involved 289 adult male participants aged between 50 and 74 years (mean = 59.0, SD = 6.1), which corresponds to the target population of the Slovenian national colorectal cancer screening programme Svit. All participants received official invitations from the programme at the time of data collection. According to Mundfrom, Shaw and Ke [22], a minimum participant-to-item ratio of 3:1 to 6:1 is considered sufficient for conducting factor analyses when commonalities are moderate to high (i.e. > 0.40). Considering that 36 items were retained after content validation, the ratio achieved in this study was approximately 8:1. Therefore, under the observed psychometric conditions, the sample size exceeded the recommended thresholds for both exploratory and confirmatory factor analyses. 

### 2.4. Ethical Considerations

Ethical approval for the study was granted by the Ethics Committee for Human Research of the University of Primorska (number: 4264-16-3/2022). All data were kept strictly confidential, and informed consent was obtained from all participants who voluntarily agreed to take part in the study.

### 2.5. Data Collection

The data collection was carried out between January and June 2023. A mixed survey approach was used, combining both online and paper-based questionnaires. The online survey was developed using the open-source platform 1KA (https://www.1ka.si/d/en) and launched in January 2023. The link to the survey was distributed via various social media channels, including Facebook and relevant community or health-related associations. In addition to the online version, paper questionnaires were made available at selected primary health centres, local community centres and health promotion events. All participants received clear information about the purpose of the study and instructions on how to complete the questionnaire, either in digital or printed form.

To ensure anonymity, no personal identifiers were collected. The final dataset was only accessible to the principal investigator to maintain confidentiality throughout the analysis. Informed consent was obtained from all participants. They voluntarily agreed to participate and confirmed that they understood the research objectives.

### 2.6. Data Analysis

The data were analysed using IBM SPSS Statistics version 29.0 (IBM Corp., Armonk, NY, USA) and Jamovi version 2.4.5 (The Jamovi Project, Sydney, Australia), an open-source statistical platform based on R. Descriptive statistics were calculated to summarise the demographic characteristics of the participants.

An exploratory factor analysis with Promax rotation was conducted to determine the factor structure of the scale. The Kaiser–Meyer–Olkin test (KMO) and the Bartlett test for sphericity were then performed to assess sample adequacy. Items with factor loadings below 0.40 were excluded. A confirmatory factor analysis and structural equation modelling (SEM) were then performed to assess the construct validity of the proposed factor structure. Model fit was assessed using the chi-square test (χ2), the Comparative Fit Index (CFI), the Tucker–Lewis Index (TLI), the Root Mean Square Error of Approximation (RMSEA) and the Standardised Root Mean Square Residual (SRMR).

The reliability of internal consistency was assessed using Cronbach’s alpha (α), with values ≥ 0.70 considered acceptable for new instruments [21]. A statistical significance level of 0.05 was used for the analyses.

## 3. Results

A total of 289 Slovenian men between the ages of 50 and 74 took part in this study. As shown in Table 1, the majority of them were employed (63.3%) and had completed at least upper secondary education (36.3%). A detailed overview of the sample characteristics is presented in Table 1.

### 3.1. Content Validity

The Item Content Validity Index (I-CVI) was calculated for each item. Items with an I-CVI ≥ 0.78 were selected for further analysis. Based on this criterion, 28 items were excluded due to insufficient content validity or clarity. The remaining 36 items resulted in an average scale Content Validity Index (S-CVI/Ave) of 0.94 for relevance and 0.91 for clarity, both above the recommended threshold of 0.90, indicating strong evidence of content adequacy and conceptual clarity of the scale items.

To assess variability in expert ratings, the coefficient of variation (CV) was computed for each item. The CV was calculated for each item to assess the variance of the expert ratings. Most items had CV values below 15%, indicating a high level of agreement between raters. No item exceeded the critical threshold of 40%, which supports the internal consistency and acceptability of item-level expert agreement.

In addition, Kendall’s W was used to assess the inter-rater agreement for all items. The analysis revealed a moderate and statistically significant level of agreement for the relevance ratings (W = 0.453, *χ*2(35) = 89.049, *p* = 0.007) and a higher, also significant level of agreement for the clarity ratings (W = 0.571, *χ*2(35) = 27.246, *p* = 0.022).

### 3.2. Construct Validity

Following the content validity analysis, construct validity was further assessed through exploratory factor analysis and confirmatory factor analysis. The suitability of the dataset for factor analysis was confirmed by the Kaiser–Meyer–Olkin (KMO) measure of sampling adequacy (KMO = 0.838) and Bartlett’s Test of Sphericity (*χ*2 = 2603.507, *p* < 0.001). An EFA was conducted using principal axis factoring with Promax rotation, retaining factors with eigenvalues greater than 1.0 and items with factor loadings ≥ 0.40. Thirteen items were removed based on these criteria, resulting in a refined 23-item scale. The five extracted factors explained a cumulative 61.9% of the total variance. An overview of the retained items, factor structure, loadings and content validity indices is provided in Table 2.

**Figure 1:**
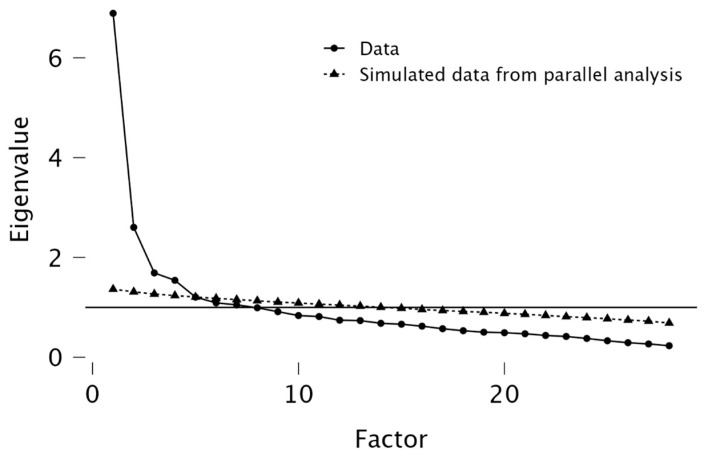
Scree plot with parallel analysis for determining the number of factors.

Exploratory and confirmatory factor analyses supported a five-factor structure of the MR–CCSS, suggesting that the scale captures five interrelated psychosocial dimensions relevant to men’s participation in colorectal cancer screening. The factors include (1) stigma, embarrassment and shame associated with participation in screening (explained variance: 29.2%); (2) denial of the need for screening and use of excuses (9.3%); (3) healthy lifestyle and positive attitude towards health (8.1%); (4) understanding the screening programme (8.0%); and (5) fear of diagnosis and disease (7.2%). Overall, the five-factor structure accounted for 61.9% of the total explained variance, supporting the multidimensional nature of the scale. The number of factors was determined based on eigenvalues ≥ 1.0, scree plot analysis (Figure 1) and parallel analysis, all suggesting a five-factor solution.

Subsequently, a CFA was performed to validate the five-factor model derived from an EFA. The model demonstrated an acceptable fit to the data, as indicated by the following fit indices: *χ*2(168) = 546, *p* < 0.001, CFI = 0.928, TLI = 0.910 and RMSEA = 0.056 (90% CI: 0.051–0.061). Standardised factor loadings ranged from 0.257 to 1.024 and were all statistically significant (*p* < 0.001), supporting the convergent validity of the constructs. Additionally, the inter-factor correlations were statistically significant, indicating meaningful relationships between the latent variables. 

Furthermore, factorial covariances were examined to assess the relationships between the five latent constructs identified in the CFA (Table 3). All inter-factor covariances were statistically significant (*p* < 0.001), with estimates ranging from 0.172 to 0.619. The strongest correlation was observed between stigma, embarrassment and shame and denial of the need for screening and excuses (*r* = 0.619), while the weakest was between denial of the need for screening and excuses and healthy lifestyle and positive attitude towards health (*r* = 0.172). These findings support the theoretical coherence of the model by indicating meaningful and distinct yet interconnected constructs.

### 3.3. Internal Consistency

Internal consistency reliability was assessed for each of the five identified factors using Cronbach’s alpha coefficient. The results indicated acceptable to high reliability for most factors: Factor 1 (α = 0.782), Factor 2 (α = 0.828), Factor 3 (α = 0.758) and Factor 5 (α = 0.833). Although Factor 4 demonstrated a slightly lower Cronbach’s alpha (α = 0.665), it was retained due to its conceptual relevance and contribution to the overall factorial structure. Previous studies have acknowledged that alpha values between 0.60 and 0.70 may be acceptable in exploratory research, especially when the factor is theoretically meaningful and supported by strong loadings in confirmatory analysis [22]. The overall MR–CCSS demonstrated good internal consistency (α = 0.863). Detailed reliability estimates are presented in Table 4.

To further explore the distribution of responses across the identified factors, a one-sample t-test was conducted to examine whether the mean scores significantly differed from the midpoint of the five-point Likert scale (i.e., test value = 3). As shown in Table 4, all factors demonstrated statistically significant deviations from the midpoint (*p* < 0.001). The mean scores for Factor 1 (stigma, embarrassment and shame) and Factor 2 (denial of the need for screening and excuses) were significantly lower than the scale midpoint, indicating general disagreement with these barriers. Conversely, higher mean scores were observed for Factor 3 (healthy lifestyle and positive attitude towards health) and Factor 4 (understanding of the screening programme), reflecting positive attitudes and knowledge about screening. The highest agreement was recorded for Factor 4 and the lowest for Factor 1. These findings support the interpretability of the factors and suggest meaningful variability in participants’ attitudes across the identified dimensions.

### 3.4. Scoring of the Men’s Response to Colorectal Cancer Screening Scale (MR–CCSS)

The MR–CCSS consists of 23 items, each rated on a 5-point Likert scale (1 = strongly disagree to 5 = strongly agree). This results in a possible total score of 23 to 115. Higher scores indicate a greater willingness and a positive attitude towards participation in colorectal cancer screening.

To determine the optimal cut-off point for identifying individuals more likely to participate in the Svit screening programme, an ROC curve analysis was conducted. The MR–CCSS showed acceptable discriminatory power with an area under the curve (AUC) of 0.702 (95% CI: 0.647–0.758, *p* < 0.001), indicating a moderate ability to discriminate between participants and non-participants in the screening programme. The optimal cut-off was determined using the Youden’s Index, with the best balance between sensitivity and specificity observed at a score of 92.5. At this cut-off, the sensitivity was 0.782 and the specificity was 0.509, suggesting that a score of 92.5 most accurately separates individuals who are likely to engage in screening from those who are not. Based on this, values ≥ 92.5 reflect a high probability of responsiveness to screening invitations [23].

The results indicated a statistically significant difference in the overall score on the MR–CCSS depending on participation in the Svit screening programme (Mann–Whitney U = 8,663.00, Z = –5.823, *p* < 0.001). Men who participated in the programme (*n* = 215, Me = 100.00, SD = 9.21) had, on average, a statistically significantly higher score on the scale compared to men who did not participate (*n* = 74, Me = 91.80, SD = 12.20). The effect size, expressed as a rank-biserial correlation (*r* = 0.421), indicates a moderate association between participation in the Svit screening programme and higher scores on the responsiveness factor scale. In addition, a statistically significant negative correlation was found between participants’ age and their MR–CCSS total score (Spearman’s ρ = –0.496, *p* < 0.001), indicating a moderately strong inverse relationship.

## 4. Discussion

This study aimed to develop and validate the Men’s Response to Colorectal Cancer Screening Scale (MR–CCSS), a gender-sensitive instrument designed to measure factors that influence men’s participation in colorectal cancer screening. The results provide initial evidence for the reliability, construct validity and discriminatory power of the scale and contribute to a better understanding of male-specific attitudes, beliefs and barriers related to participation in screening.

In the following discussion, each construct is interpreted as a specific dimension of the MR–CCSS scale that provides insight into its theoretical and practical implications. Together, these constructs form the validated structure of the instrument.

The first construct, “Stigma, embarrassment and shame associated with participation in screening”, proved to be the most dominant factor on the scale and explained the largest proportion of the variance. This indicates the central role that psychosocial discomfort, and in particular the perception of colorectal health as a private or taboo subject, plays in men’s willingness to attend screening. Previous research has consistently shown that the embarrassment of having a sample taken, reluctance to talk about bowel problems, and fear of potential diagnoses prevent men from being screened for colorectal cancer [24]. These barriers often amplify traditional masculinity norms, which tend to emphasise stoicism, emotional restraint and self-confidence and may present preventative health behaviours as a sign of weakness [25,26]. More recent findings further support this explanation. González-Flores et al [15] found that men frequently suffer from procrastination avoidance and fundamental fatalism, often believing that the absence of symptoms justifies non-attendance in an examination. Their study also found that men are more likely than women to perceive colorectal cancer screening as intrusive and shameful, with stigma playing a key role in delaying or avoiding participation.

The second construct of the MR–CCSS, “Denial of the need for screening and use of excuses”, captures cognitive minimisation strategies and everyday rationalisations that men use to justify not attending colorectal cancer screening. Typical examples include beliefs such as “I feel healthy”, “I have no symptoms” or “I’m too busy”, which reflect a lower perceived susceptibility and reduced personal relevance of screening [27]. This pattern is consistent with previous research showing that perceived susceptibility is one of the strongest predictors of preventive health behaviours, as observed in the Health Belief Model [28]. Men are particularly prone to underestimating risk and thinking avoidantly or dismissively when confronted with asymptomatic diseases such as colorectal cancer [9]. It has also been researched [15] that men tend to postpone decisions about screening due to fatalistic beliefs, a perceived invincibility and a reactive rather than preventative utilisation of health services. Such attitudes are often supported by societal expectations that discourage men from recognising their vulnerability or prioritising routine health check-ups. Studies have also shown that men are more likely than women to postpone medical consultations until symptoms become severe, suggesting a general tendency towards reactive rather than proactive health behaviours [29].

The third construct of the scale, “Healthy lifestyle and positive attitude towards health”, reflects an internalised sense of responsibility for personal well-being, which is characterised by health-promoting behaviours such as regular physical activity, a balanced lifestyle and an orientation towards preventive healthcare. Individuals who score highly on this factor are more likely to view their health as an asset that needs to be continuously maintained and are therefore more receptive to invitations for preventive check-ups. This is consistent with evidence showing that health-conscious people are more likely to engage in preventive behaviour, including cancer screening [30,31]. Studies have also shown that a positive attitude towards health, such as the view that health is a personal responsibility, increases the willingness to participate in organised screening programmes [32]. Furthermore, this construct is consistent with the concept of ‘internal health locus of control’, where individuals believe that their actions directly affect their health outcomes [33]. Men with such an orientation are more likely to recognise the value of screening and take the initiative to maintain their wellbeing, in contrast to men with an external or fatalistic view [34].

The fourth construct of the scale “Understanding the screening programme” reflects participants’ knowledge, understanding and confidence in Svit’s colorectal cancer screening programme. This includes knowledge of the objectives and procedures of the programme as well as perceived clarity of communication materials such as invitation letters and instructions for taking stool samples. Higher scores on this factor indicate better information processing, which is essential for informed decision making and active participation. Numerous studies have shown that limited understanding of the purpose and procedures of colorectal cancer screening is a major barrier to participation, particularly among individuals with low health literacy [35]. In addition, men’s screening behaviour is significantly influenced by their trust in the healthcare system and their belief in the relevance of the information provided. If messages are perceived as vague, too technical or not relevant, men may withdraw or delay their participation [36]. Conversely, trust in public health facilities and clear, understandable messages have been shown to improve the uptake of screening [37].

The last construct of the scale “Fear of diagnoses and diseases” captures the emotional and existential reactions to the possibility of discovering a serious disease, especially cancer, through screening. High scores on this factor reflect anxiety, uncertainty and avoidance behaviour triggered by the fear of a life-changing diagnosis. This pattern of behaviour has been frequently described in previous research where fear of cancer has been shown to be a significant psychological barrier to screening participation [38]. Individuals often report that they would rather not know or avoid screening altogether in order to delay or avoid the distressing emotional impact of a potential diagnosis [27]. Other studies have shown that fear-based avoidance is a dominant theme among men, who may perceive confirmation of a diagnosis as a threat not only to their physical health but also to their role and identity. This is consistent with the concept of ‘anticipated regret’, where individuals fear the consequences of realising they have cancer more than the disease itself, particularly in cases where symptoms are not yet present [39].

Understanding the unique psychological and social experiences of men in the context of health screening is essential for truly person-centred care. The MR–CCSS scale allows us to look beyond demographic data and explore the deeper motivations and hesitations that influence health-related decisions. In everyday healthcare practice, this can be especially useful for nurses, educators and public health workers trying to communicate more effectively and support men to make informed decisions.

### Limitations

Although the MR–CCSS showed good psychometric properties, several limitations should be considered when interpreting the results. First, despite efforts to include both online and paper-based data collection methods, the sample may have been influenced by self-selection bias, as individuals more interested in health-related topics may have been more likely to participate. Secondly, although the scale is based on qualitative data and expert opinion, the cross-sectional design limits the ability to draw conclusions about causality. Future research should consider longitudinal validation and test–retest reliability to assess the scale’s stability over time. Even though the scale is based on qualitative data and validated by experts, it was developed and tested in the Slovenian cultural and health context. Health beliefs, stigmatisation, masculinity norms and trust in screening programmes can vary considerably in different cultures. Therefore, the MR–CCSS should be culturally adapted and validated in other countries and populations to ensure its broader applicability and relevance. Finally, an optimal cut-off score was identified using the ROC analysis, but further validation in different community settings is required to confirm the predictive value of the scale in practice.

## 5. Conclusions

The five-factor MR–CCSS is a valid and reliable instrument for assessing psychosocial and behavioural factors that influence men’s participation in colorectal cancer screening. It offers valuable insights into men’s perceptions, emotions and motivations related to screening, aspects that are often overlooked in standard health promotion efforts. While some men may be reluctant due to fear, uncertainty or shame, others may not consider screening important or necessary. The MR–CCSS helps to uncover these differences and provides healthcare professionals with a clearer understanding of who might benefit from additional support, encouragement or tailored information. Recognising these perspectives creates opportunities for more meaningful conversations and health education that directly addresses men’s concerns. Ultimately, the newly developed scale supports a more equitable approach to public health through facilitating informed decision making and helping to identify men who are at greater risk of not engaging.

## Figures and Tables

**Table 1:** Characteristics of the participants (*n* = 289).

Characteristic	*n*	%
Employment status:		
Employed	183	63.3
Self-employed	34	11.8
Retired	70	24.2
Unemployed	2	0.7
Level of education:		
Primary education (ISCED 1/SOK II)	20	6.9
Lower vocational education (ISCED 2–3C/SOK III–IV)	59	20.4
Upper secondary education (ISCED 3A/SOK V)	105	36.3
Short-cycle higher education/professional diploma (SOK VI/1)	42	14.5
Bachelor’s degree (Bologna 1st cycle/SOK VI/2)	32	11.1
Master’s degree (Bologna 2nd cycle/SOK VII, VIII/1)	36	12.5
Doctoral degree (Bologna 3rd cycle/SOK VIII/2)	7	2.4
Participation in the Svit Programme (stool sample submitted)		
Yes	221	76.5
No	68	23.5

Note. Education levels are presented in accordance with the Slovenian Qualifications Framework (SOK) and aligned with the Bologna structure for international comparison.

**Table 2:** Overview of the retained items with factor loadings, significance levels and content validity indices.

Items		Factor Loadings	z-Value	*p*	I-CVI (R, C)
Factor 1	I hesitate to talk about digestive problems.	0.820	23.47	<0.001	1.00
	I believe that it is inappropriate to talk about the bowel and the anus.	0.783	14.74	<0.001	0.91
	I don’t talk to anyone about problems involving intimate parts of the body.	0.688	20.46	<0.001	1.00
	I am afraid that people will think I am homosexual if I undergo a colonoscopy.	0.682	14.04	<0.001	1.00
	I didn’t take part in the Svit programme because I didn’t understand the language.	0.619	14.97	<0.001	0.91
	I think women have a higher risk of developing colorectal cancer.	0.580	12.20	<0.001	0.91
	Colorectal cancer only affects people who sit too much.	0.548	14.20	<0.001	0.91
	In our family, it is the woman who looks after the health of the family members.	0.508	9.80	<0.001	1.00
	I think the Svit programme is unnecessary because I already know the signs of colorectal cancer.	0.408	11.60	<0.001	0.91
Factor 2	I didn’t respond to the invitation to the Svit programme because I don’t think I have any health problems.	0.892	24.24	<0.001	1.00
	I don’t have time to take part in screening programmes.	0.878	24.00	<0.001	0.91
	It would be easier for me if my GP invited me to the Svit programme and the nurse explained everything to me.	0.678	20.59	<0.001	0.91
	I didn’t fully understand the invitation to the Svit screening programme.	0.528	15.80	<0.001	1.00
	I don’t take care of my own bowel movements; if necessary, the doctor sends me for a check-up.	0.408	19.50	<0.001	0.91
Factor 3	I make sure I lead a healthy lifestyle.	0.870	22.07	<0.001	0.82
	I believe that my lifestyle is healthy.	0.805	18.91	<0.001	0.91
	I make sure I am physically active for at least 30 min every day.	0.649	15.80	<0.001	0.91
Factor 4	I know what screening programmes are.	0.840	16.12	<0.001	0.91
	I know what a colonoscopy is.	0.647	10.80	<0.001	0.91
	I know the purpose of the Svit screening programme.	0.527	18.72	<0.001	1.00
	I know the risk factors for developing colorectal cancer.	0.513	11.45	<0.001	0.91
Factor 5	I am afraid that the test will reveal something that could lead to death.	0.828	19.37	<0.001	1.00
	I am afraid that cancer will be found if I take part in the Svit programme.	0.787	16.38	<0.001	1.00

Note. Accumulated total explained variance = 70%. Bartlett’s Test of Sphericity: *χ*2 = 2603.507, *p* < 0.0001; Kaiser–Meyer–Olkin value = 0.838; SD—Standard Deviation; Factor 1—stigma, embarrassment and shame associated with participation in screening; Factor 2—denial of the need for screening and use of excuses; Factor 3—healthy lifestyle and positive attitude towards health; Factor 4—understanding the screening programme; Factor 5—fear of diagnoses and disease; Factor Rotation: Promax with Kaiser normalisation; rating is based on five response categories, ranging from 1—strongly disagree to 5—strongly agree; I-CVI (R, C)—Items Content Validity Index (Relevancy, Clarity).

**Table 3:** Factorial covariances in the structural equation model.

Factors	Estimate	Std.Error	z-Value	*p*	95% Confidence Interval	
					Lower	Upper
Stigma, embarrassment and shame ↔ Denial of the need for screening and excuses	0.619	0.013	46.875	<0.001	0.593	0.645
Stigma, embarrassment and shame ↔ Healthy lifestyle and positive attitude towards health	0.197	0.014	14.195	<0.001	0.170	0.244
Stigma, embarrassment and shame ↔ Understanding the screening programme	0.380	0.013	30.252	<0.001	0.356	0.405
Stigma, embarrassment and shame ↔ Fear of diagnosis and disease	0.440	0.014	30.577	<0.001	0.412	0.468
Denial of the need for screening and excuses ↔ Healthy lifestyle and positive attitude towards health	0.172	0.015	11.498	<0.001	0.143	0.201
Denial of the need for screening and excuses ↔ Understanding of the screening programme	0.455	0.013	34.931	<0.001	0.420	0.480
Denial of the need for screening and excuses ↔ Fear of diagnosis and disease	0.454	0.014	31.337	<0.001	0.425	0.482
Healthy lifestyle and positive attitude towards health ↔ Understanding of the screening programme	0.261	0.016	16.619	<0.001	0.230	0.292
Healthy lifestyle and positive attitude towards health ↔ Fear of diagnosis and disease	0.215	0.019	11.385	<0.001	0.178	0.251
Understanding of the screening programme ↔ Fear of diagnosis and disease	0.245	0.014	16.884	<0.001	0.216	0.273

Note. Items related to Factors 1, 2 and 4 were reverse coded to ensure that higher values uniformly represented favourable attitudes towards screening. This adjustment resulted in consistently positive and interpretable inter-factor correlations.

**Table 4:** Descriptive statistics, internal consistency and one-sample *t*-test results for the five factors and overall MR–CCSS.

Factors/Subscales	*n*	Mean	SD	Cronbach α	95% Confidence Interval		*p*
					Lower	Upper	
Stigma, embarrassment and shame	9	1.51	0.485	0.782	1.47	1.54	<0.001
Denial of the need for screening and excuses	5	1.57	0.710	0.828	1.52	1.62	<0.001
Healthy lifestyle and positive attitude towards health	3	3.79	0.682	0.758	3.74	3.84	<0.001
Understanding the screening programme	4	4.06	0.687	0.665	4.01	4.11	<0.001
Fear of diagnosis and disease	2	1.88	1.042	0.833	1.80	1.95	<0.001
Men’s Response to Colorectal Cancer Screening Scale (MR–CCSS)	23	2.29	0.348	0.863	2.27	2.32	<0.001

Note. M*dn*—Median; SD—Standard Deviation.

## Data Availability

The data are available from the corresponding author upon reasonable request, as the participants were assured that it would remain confidential.
